# Predictors of Colorectal Cancer Survival in Golestan, Iran: A Population-based Study

**DOI:** 10.4178/epih/e2013004

**Published:** 2013-06-20

**Authors:** Mohammad Aryaie, Gholamreza Roshandel, Shahryar Semnani, Mohsen Asadi-Lari, Mohsen Aarabi, Mohammad Ali Vakili, Vahideh Kazemnejhad, Seyed Mehdi Sedaghat, Masoud Solaymani-Dodaran

**Affiliations:** 1 Golestan Research Center of Gastroenterology and Hepatology, Golestan University of Medical Sciences, Gorgan, Iran.; 2 Department of Epidemiology and Oncopathology Research Centre, Iran University of Medical Sciences, Tehran, Iran.; 3 Department of Public Health, Golestan University of Medical Sciences, Gorgan, Iran.; 4 5th of Azar Hospital, Golestan University of Medical Sciences, Gorgan, Iran.; 5 Minimally Invasive Surgery Research Center, Rasoul Akram Hospital, Tehran University of Medical Sciences, Tehran, Iran.; 6 Department of Epidemiology and Biostatistics, Public Health Faculty, Tehran University of Medical Sciences, Tehran, Iran.

**Keywords:** Colorectal cancer, Stage, Survival

## Abstract

**OBJECTIVES:**

We aimed to investigate factors associated with colorectal cancer survival in Golestan, Iran.

**METHODS:**

We used a population based cancer registry to recruit study subjects. All patients registered since 2004 were contacted and data were collected using structured questionnaires and trained interviewers. All the existing evidences to determine the stage of the cancer were also collected. The time from first diagnosis to death was compared in patients according to their stage of cancer using the Kaplan-Meir method. A Cox proportional hazard model was built to examine their survival experience by taking into account other covariates.

**RESULTS:**

Out of a total of 345 subjects, 227 were traced. Median age of the subjects was 54 and more than 42% were under 50 years old. We found 132 deaths among these patients, 5 of which were non-colorectal related deaths. The median survival time for the entire cohort was 3.56 years. A borderline significant difference in survival experience was detected for ethnicity (log rank test, p=0.053). Using Cox proportional hazard modeling, only cancer stage remained significantly associated with time of death in the final model.

**CONCLUSIONS:**

Colorectal cancer occurs at a younger age among people living in Golestan province. A very young age at presentation and what appears to be a high proportion of patients presenting with late stage in this area suggest this population might benefit substantially from early diagnoses by introducing age adapted screening programs.

## INTRODUCTION

Colorectal cancer (CRC) is the most common cancer among gastrointestinal malignancies and the fourth leading cancer-related death in the world [1]. CRC is the fourth most common cause of cancer after stomach, esophagus and lung in men and the fifth most common after breast, esophagus, blood and stomach in women in the Golestan region of Iran [2], with an increasing trend line [3]. About 40% of CRC cases are diagnosed in the preliminary stages, with a high five-year survival rate, while those diagnosed in the metastatic phase have only a 5% chance of living beyond the fifth year [4]. The median survival rate for colorectal cancer in Iran has been reported to be 3.5 years [5].

Available estimates of colorectal cancer survival in Iran [3,5,6] are hospital-based. However, in order to have a more accurate assessment of disease impact on health and more relevant estimates that can be used in health care planning, there is a need for population-based studies. Hospital based figures may represent a socio-economically distinct population of those who have had sufficient access to health care resources. Golestan's population-based cancer registry provided the opportunity for us to address this important question in a population-based setting.

We have therefore used a cohort of patients with colorectal cancer registered with the Golestan cancer registry and looked at predictors of survival in this population.

## MATERIALS AND METHODS

This research was approved by the Ethics Committee of the Tehran University of Medical Science, Golestan University of Medical Science and the Golestan Research Center of Gastroenterology and Hepatology (GRCGH).

This study was conducted in the Golestan province area, which is more than 20,367 km^2^ and has a population of about 1,650,000 according to the latest census (2004). The province shares 320 km of water and terrestrial borders with Turkmenistan and is located in the southeast littoral of the Caspian Sea in northern Iran. Forty percent of the population living in Golestan province are Persians. Other ethnic types include Turkmen (32%), Sistanis (also called Zabolis) (15%) and Azeri Turks (5%) [7]. Turkmen people in Golestan province are a branch of Asia-pacific Turkmen who live a traditional life, and intra-familial marriages are prevalent among them. The Mediterranean style climate makes agriculture one of the major occupations among the people living in this region.

We retrospectively built our cohort from all registered colorectal cancer cases over 4 years (2004 to 2008) in the GRCGH, which has been accepted as a voting member of the International Agency for Research on Cancer. GRCGH collects data on gender, date of birth, age at diagnosis and histology of tumors for all newly diagnosed cancer patients in Golestan province.

All cohort members were contacted at their home addresses, and information on their current status as well as demographics and available outpatient records were collected. Structured questionnaires were used for this purpose. A group of 12 trained interviewers covered the entire province, consisting of 12 cities and their surrounding rural areas. Interviewers were selected from local healthcare staff with local knowledge, and special care and training were provided to deal with situations where the patient had passed away. Information was collected from closest relatives in these cases. Causes of all deaths were matched with death registry information. All relevant medical institutions were also contacted, including private and public hospitals, pathology departments, diagnostic laboratories and local health centers to gather information regarding their colorectal cancers.

The follow-up period started at the time of first diagnosis of colorectal cancer and ended at the time of interview for those who were alive at that time. For those who had died or were lost to follow-up, the date of death or the latest date when the patient's status was known were used as the end of follow-up date. No information was collected on those who could not be traced (e.g., incorrect addresses or those who had abandoned their address). Time and cause of death were determined by interviewing first degree relatives and the available outpatient records. Losses to follow-up and deaths not due to colorectal cancer were regarded as censored.

We used a summary staging developed by the Surveillance, Epidemiology and End Results (SEER) program for population-based cancer registries [8]. We collected and re-examined all available medical evidences including CT-scan, MRI, sonography, colonoscopy reports, lab results such as fecal occult blood test and pathological reports by the gastroenterologist and pathologist on the research team. The stage of the tumor was reassessed in all but 18 patients and re-classified as in situ, localized, regional or tumors with distant metastasis [9].

We calculated crude mortality rates by dividing the total number of deaths in our cohort by the total person time follow-up. Mortality due to colorectal cancer and its 95% confidence interval (CI) were also calculated in the same manner. The time from diagnosis till death were compared in patients according to their different characteristics using the Kaplan-Meier method and log rank testing. Cox proportional hazard models were built to examine their survival experience by taking into account other covariates including age, gender, ethnicity, place of residence, and complementary insurance. Hazard ratios (HRs) and their 95% CIs were calculated. Proportional hazard assumptions for each model were checked using log minus log plots and a proportional hazard test. Data were analyzed using SPSS version 16.0 (SPSS Inc., Chicago, IL, USA) and a p-value less than 0.05 was considered as significant.

## RESULTS

We analyzed 227 cases of colorectal cancer in our cohort, out of a total of 345 registered cases. Overall 132 deaths were identified among these patients, 2 of which were accidental and 3 of which were due to heart attack related deaths, over 7,484 months of follow-up. The average follow-up time was 33 months. The median age at diagnosis was 54 years (range, 14 to 85 years) with more than 42% less than 50 years of age and a male to female ratio of 1.55. There were 42, 105, and 62 patients in the localized, regional, and distant metastasis stages, respectively and the tumor stage in 18 patients was unknown. During the study period, 61 (98.4%) patients with distant metastasis, 48 (45.7%) patients in the regional stage and 9 (21.4%) patients in the localized stage died due to CRC. The detailed patient demographic characteristics in relation to tumor stage are summarized in Table 1.

**Figure 1:**
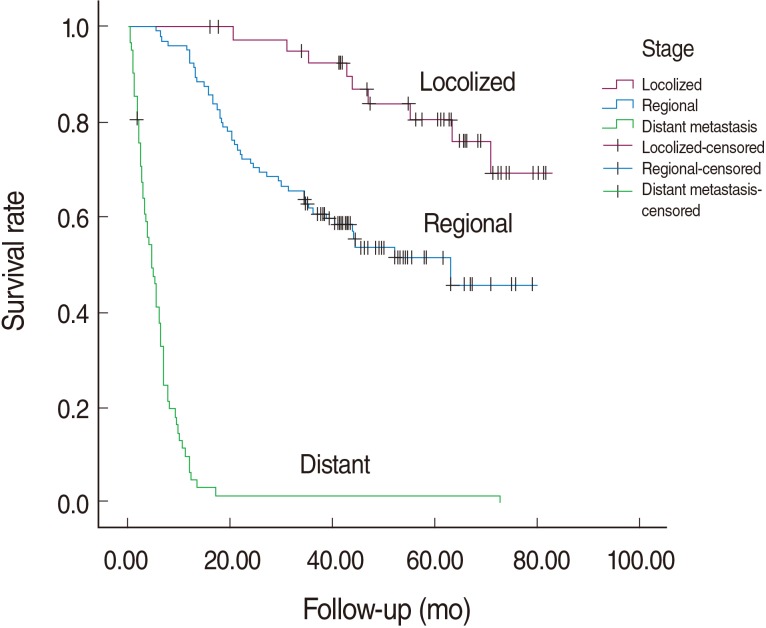
Overall survival of colorectal cancer cases in Golestan, Iran by stage.

The median survival time for colorectal cancer was 3.56 years in our cohort. The median survival time in relation to tumor stage is shown in Table 2. Before adjusting for potential confounders, the survival rate in patients of Turkmen ethnicity was lower than in non-Turkmen (log rank test, p-value=0.053; HR, 48, 95% CI, 0.99-2.21) and in those without complementary insurance compared with those who had such insurance (log rank test, p-value=0.016; HR, 1.83; 95% CI, 1.12-2.99). The differences in survival rate between men and women and for age groups at diagnosis were not statistically significant. Kaplan-Meier curves for different tumor stages are plotted in Figure 1. One, five and six-year survival rates by tumor stage were 100, 80.6 and 69.2 percent for the localized stage and 92.4, 51.5 and 45.8 percent in the regional stage. Almost all patients with distant metastases had died before the first year of follow-up.

After adjusting for potential confounders including age groups, gender, ethnicity, living area and possession of complementary insurance schemes, patients in the distant metastasis and regional stages were 55.9 (95% CI=1.66-7.99) and 3.43 (95% CI=1.66-7.99) times more likely to die during the study period, respectively, compared with patients in the localized tumor stage (Table 3). There were no serious violations of proportional hazard assumptions in checking the model.

## DISCUSSION

We found that colorectal cancer stage is the most important independent predictor of survival among colorectal cancer patients in the Golestan region of Iran. Ethnicity and possessing a complementary health insurance scheme, although significantly associated with survival in the unadjusted analysis, were no longer predictors when other potential confounders were taken into account. We also found that our colorectal cancer cohort represented a relatively youthful population compared to other national and international estimates [5,9-13].

Our finding of staging to be an independent and most important predictor of survival is consistent with findings from others [6,9,10]. We used a staging method introduced by the SEER program called summary staging. Even though SEER summary staging is a tool used in population studies and not normally in clinical settings, its validity for this purpose has already been demonstrated [14].

Near half of the patients in our study were less than fifty years of age. This figure is relatively high compared to other national and international estimates [6,9-13]. Possible genetic and environmental factors could play a role, including the low socioeconomic status of the population and subsequent lower consumption of fresh fruits and vegetables. A similar low age of incidence of colorectal cancer has been reported from Egypt [15]. Exposure to chemicals used in agriculture and, in more recent studies, genetic changes have also been suggested as possible explanations [16]. Since agriculture is one of the main jobs in Golestan province and around 25% of cases were farmers, adverse environmental factors consisting of indiscriminate use of herbicides, pesticides and fertilizers, or unrestricted antibiotics and hormones, could also explain this finding.

We found that age at diagnosis was not a significant prognostic factor for colorectal cancer. Contrary to our findings, some studies have suggested that age at diagnosis significantly affects a patient's outcome [6,9,17]. Those suggesting that age is a prognostic factor did not report a significant difference in survival rate across age groups except for poor survival in the elderly [5]. This may be due to poor general health and the difficulties of cancer therapy in the elderly [12]. Young patients are more likely to be diagnosed in later stages because the suspicion of malignancy might not be at the top of their differential diagnosis list; therefore their survival rates may be relatively worse [18,19].

Our study did not show any difference in survival experience between men and women. It has been hypothesized that hormonal factors, immune function [20] and steroids in particular may cause better survival and protection for women [21]. However, gender did not seem to influence overall survival in this analysis. Our findings have been supported by some other studies [1,6,12,17,22], although heated controversies exist [23-26]; in some studies an association was detected only in univariate analysis [5,13].

Race/ethnicity have been previously used as measures of socioeconomic status [27-29]. We found a significant association between ethnicity and survival in univariate analysis; however, it disappeared after adjusting for other potential confounders. Recent investigations conducted in both rural and urban Gonbad parts of Golestan province, with a predominant Turkmen population, indicated that the rates of overweight (body mass index [BMI]>25), and obesity (BMI>30) were 60% and 25%, respectively. This may explain the increase in risk of colorectal carcinoma in men and women in this area. One study also showed poor access to safe drinking water, electricity, and natural gas before the 1970s [30].

We did not find a statistically significant difference in survival among those who had complementary health insurance schemes compared with those who did not, despite the initial observed differences in the unadjusted analysis. The usual healthcare insurance in Iran does not cover all the costs of services; it is estimated to cover only 30 to 40%. This is why complementary health insurance has become an important addition to standard insurance plans in Iran and is expected to play an important role in helping patients seek medical advice early on during the course of their illness. This in turn could have a significant impact on timely treatment and survival rates. However, our finding might be due to the relatively young age of these complementary schemes, particularly in Golestan province (just a few years) and we expect to see their effect more clearly in the future as they become more established and widespread.

We were unable to locate about a third of the original cohort of registered patients, which could suggest the possibility of a selection bias. We therefore compared existing information on all registered patients, including age at diagnosis, place of residence, gender, and year of diagnosis in those who had been lost to follow-up with the rest of the cohort (Table 4). The two groups had similar ages at diagnosis and year of diagnosis. Those who were lost to follow-up, however, were more likely to be women and be from urban areas (85% were from the two largest cities in the province).

The findings from the current study indicate that early diagnosis could increase survival considerably and reduce costs to health services. Other studies have also shown that the majority of Iranian subjects had poor knowledge about colorectal cancer screening [30]. Therefore this supports the case for screening programs, increasing population awareness of symptoms and/or improving access to diagnostic facilities.

In summary, our study showed that cancer stage is the most important predictor of survival in the relatively young colorectal cancer patients in Golestan province, as well as revealing a high proportion of patients presenting at late stage in this area, supporting the case for serious consideration of a nationwide age-adapted colorectal cancer screening program. This population might also benefit substantially from early diagnosis by increasing awareness and improving access to diagnostic facilities.

## Figures and Tables

**Table 1:**
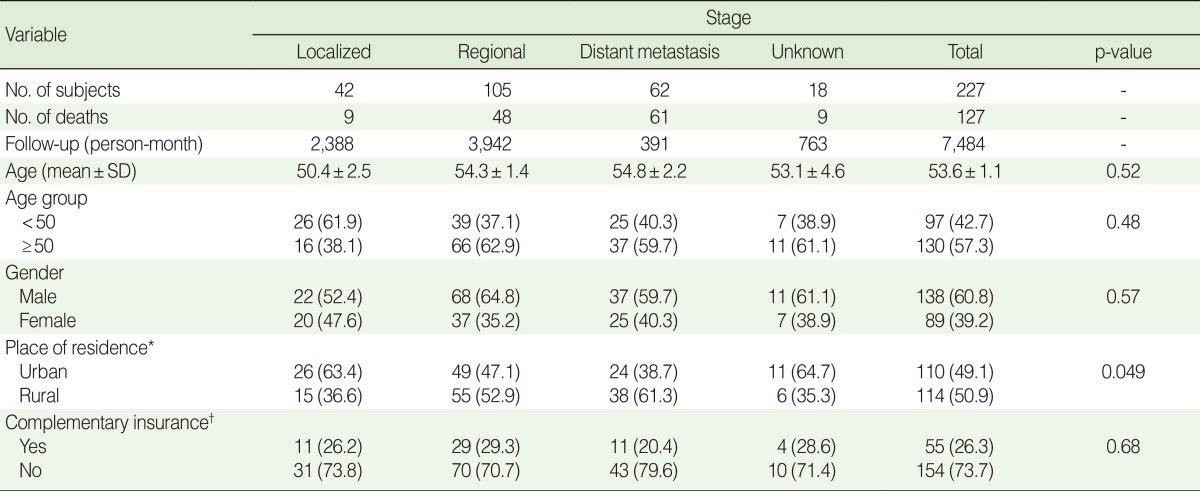
Characteristics of study subjects in relation to tumor stage

Values are presented as number (%). SD, standard deviation. ^*^There were three missing cases for place of residence; ^†^There were eighteen missing cases for complementary insurance.

**Table 2:**
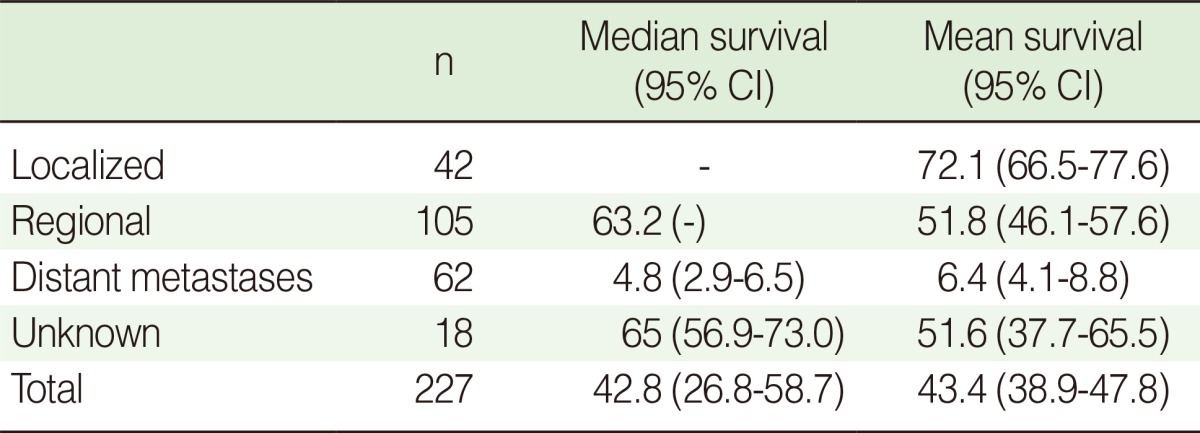
Mean and median survival of colorectal cancer patients according to stage

CI, confidence interval.

**Table 3:**
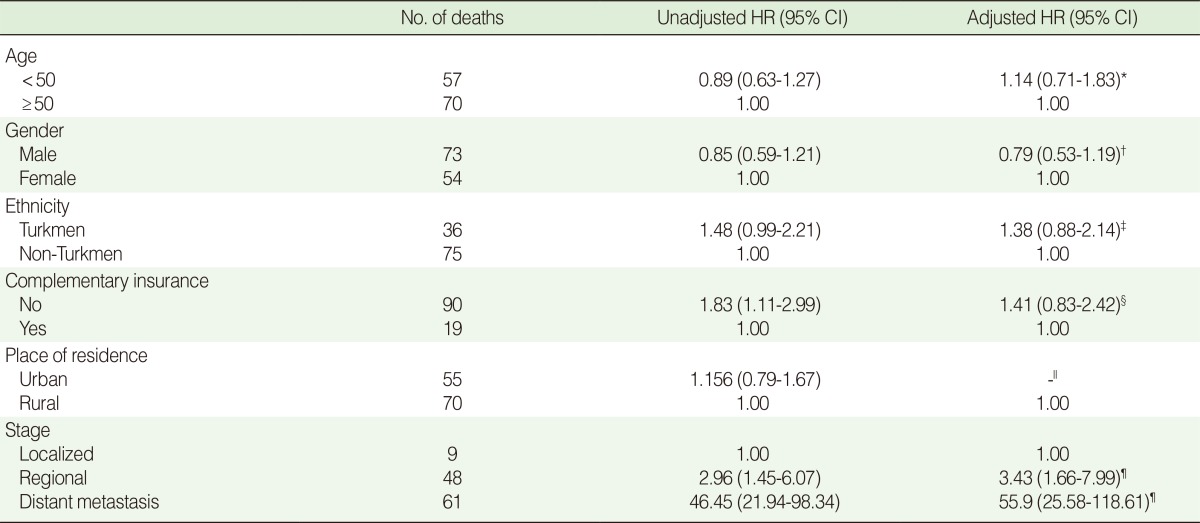
Adjusted and unadjusted colorectal cancer survival according to patient characteristics

HR, hazard ratio; CI, confidence interval. ^*^Adjusted for gender, complementary insurance, ethnicity, stage; ^†^Adjusted for age, complementary insurance, ethnicity, stage; ^‡^Adjusted for age, gender, ethnicity, stage; ^§^Adjusted for age, gender, complementary insurance, stage; ^∥^Not included in the model; ^¶^Adjusted for age, gender, complementary insurance, ethnicity.

**Table 4:**
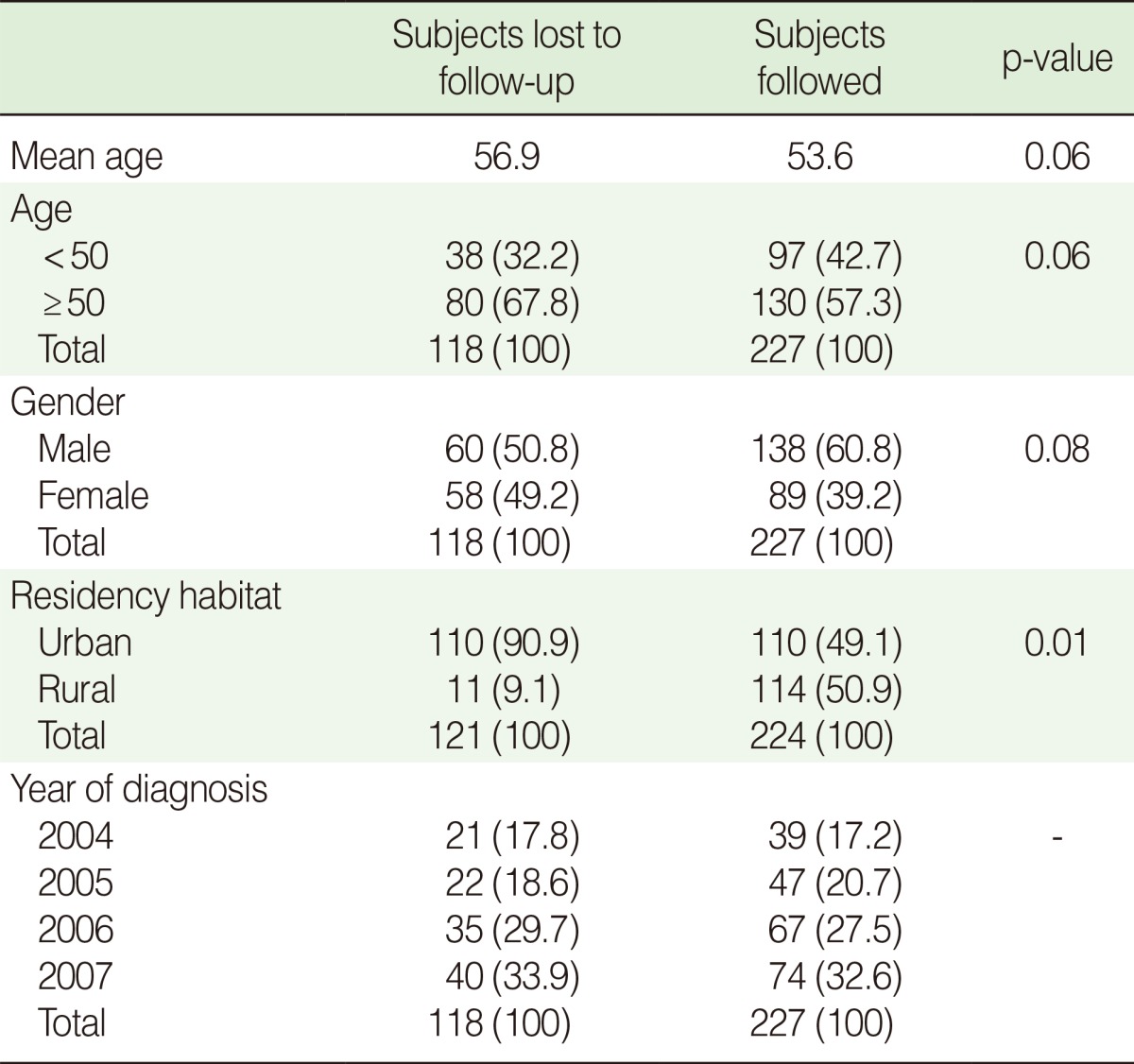
Comparison of baseline data in Golestan population-based cancer registry between follow-up and lost to follow up patients

Values are presented as number (%).
